# Analysis of lncRNA, miRNA and mRNA-associated ceRNA networks and identification of potential drug targets for drug-resistant non-small cell lung cancer

**DOI:** 10.7150/jca.40729

**Published:** 2020-03-05

**Authors:** Xiangzhen Kong, Shousen Hu, Yongliang Yuan, Yue Du, Zijia Zhu, Zhizhen Song, Shanshan Lu, Chang Zhao, Dan Yan

**Affiliations:** 1 Department of Pharmacy, the First Affiliated Hospital of Zhengzhou University, Zhengzhou, China.; 2 Henan Key Laboratory of Precision Clinical Pharmacy, Zhengzhou University, Zhengzhou, China.; 3 Department of Otolaryngology Head and Neck Surgery, the First Affiliated Hospital of Zhengzhou University, Zhengzhou, China.

**Keywords:** non-small cell lung cancer, competing endogenous RNA network, drug resistance, RNA sequencing

## Abstract

**Background**: Drug resistance to chemotherapeutic drugs or targeted medicines is an obstacle encountered in the treatment of non-small-cell lung cancer (NSCLC). However, the mechanisms of competing endogenous RNA (ceRNA) on the drug resistance in NSCLC are rarely reported. In this paper, the comprehensive expression profiles of lncRNAs and mRNAs in drug-resistant NSCLC cells were obtained by RNA sequencing.

**Methods**: The dysregulated lncRNAs, miRNAs and mRNAs in drug-resistant NSCLC cell lines were identified by RNA-sequencing and bioinformatics methods.

**Results**: A total of 39 dysregulated lncRNAs and 650 dysregulated mRNAs were identified between drug-resistant NSCLC cell lines and their parental cell lines. Additionally, 33 lncRNA-miRNA-mRNA pathways in the ceRNA network in drug-resistant NSCLC were constructed through bioinformatics methods and ceRNA regulatory rules. These comprised 12 dysregulated lncRNAs, five dysregulated miRNAs, and eight dysregulated mRNAs. In addition, lncRNA ATP2B1/miR-222-5p/TAB2 and lncRNA HUWE1/miR-222-5p/TAB2 were identified as potential ceRNA networks involved in drug resistance to NSCLC.

**Conclusions**: The current study provides a promising therapeutic strategy against the lncRNA-miRNA-mRNA ceRNA regulatory network for NSCLC treatment and deepens our comprehension of the ceRNA regulatory mechanisms related to drug resistance to NSCLC.

## Introduction

Non-small-cell lung cancer (NSCLC) accounts for approximately 85% of lung cancer, which is one of the most common malignant cancers in the world, and is characterized by high morbidity and mortality 1. Current effective therapies for NSCLC patients include surgical resection, chemotherapy, targeted therapy and immunotherapies 2,3. However, despite improvements in NSCLC therapy over the last few decades, the long-term survival rate of patients with NSCLC is still 20%-35% 4. To a certain extent, the treatment of NSCLC may fail due to resistance to chemotherapeutic drugs or targeted medicines.

Various studies have proposed that somatic activating mutations, dysregulation of drug transport, and disorders of cell apoptosis pathways or cell cycle regulation are common mechanisms of drug resistance 5,6. However, there are almost no available methods for effectively overcoming drug resistance at present. Thus, there is an imperative need to clarify novel mechanisms of drug resistance in NSCLC. Therefore, it is necessary to explore the molecular differences between drug-resistant NSCLC cell lines and their parental cell lines to identify new potential targets for the treatment of drug resistance in NSCLC, as well as to improve the effectiveness of treatments for this disease.

The competing endogenous RNA (ceRNA) mechanism stated that the transcripts of non-coding RNAs like long non-coding RNAs (lncRNAs), can act as natural microRNAs (miRNAs) sponges through competitively binding to miRNA response elements (MREs) on target mRNAs to inhibit their functions 7. In recent years, this ceRNA regulatory network has received considerable attentions, and many have studied the molecular mechanisms related to the occurrence and progression of tumors 8,9. However, there are limited researches focused on the mechanisms of ceRNAs in NSCLC drug resistance.

To explore the potential mechanisms of ceRNAs in NSCLC drug resistance, we detected the expression levels of lncRNAs, miRNAs and mRNAs in the drug-resistant NSCLC cell lines (A549/DDP and HCC827/GR) and their parent cell lines (A549 and HCC827) by RNA sequencing. We then carried out a comprehensive analysis of the dysregulated lncRNAs, miRNAs, and mRNAs using bioinformatics methods. Finally, based on the bioinformatics results and the ceRNA regulatory rules, a lncRNA-miRNA-mRNA network was constructed among the dysregulated lncRNAs, miRNAs, and mRNAs. This paper might provide new insights into the mechanisms of drug resistance and might have certain clinical significance for further research in this area.

## Materials and Methods

### Cell lines and culture conditions

A549 cells were obtained from Shanghai Meixuan Biological science and technology LTD, and HCC827 cells were kindly provided by Stem Cell Bank, Chinese Academy of Sciences. A549 and HCC827 cells were grown in RPMI-1640 (Hyclone, MA, USA) supplemented with 10% fetal bovine serum (Gibco, NY, USA) and penicillin/streptomycin. The cisplatin-resistant A549/DDP cells and the gefitinib-resistant HCC827/GR cells were generated by gradient dose treatment for almost six months. Drugs were added to maintain resistance and were withdrawn one week before the experiment.

### RNA isolation and purification

Total RNA from the cell lines was extracted by Trizol reagent (Invitrogen, CA, USA) according to the instruction. The quantity and purity of total RNA were measured by Bioanalyzer 2100 and RNA 6000 Nano LabChip Kit (Agilent, CA, USA) with a RIN number ≥8.

### LncRNA and mRNA sequencing

First, ribosomal RNA was depleted from approximately 10 μg of total RNA by the Epicentre Ribo-Zero Gold Kit (Illumina, CA, USA). And then, the rRNA-depleted RNA was broken into small pieces using divalent cations under an elevated temperature and then reverse-transcribed to create the final cDNA library by the mRNA-Seq sample preparation kit (Illumina, CA, USA). Then, paired-end sequencing (300 ± 50 bp) was performed on Illumina X10 (LC Sciences, USA) according to the vendor's recommended protocol.

### Transcript assembly

Adaptor contamination, low-quality bases, and undetermined bases were removed using Cutadapt, and the quality of sequencing was verified by FastQC (http://www.bioinformatics.babraham.ac.uk/projects/fastqc/). The reads of each sample were mapped to the human genome by Bowtie 2 10 and topaht2 11 and were constituted by StringTie 12. All transcriptomes of samples were combined to regenerate an integrated transcriptome using Perl scripts. After the ultimate transcriptome was generated, the expression level of all transcripts was estimated by StringTie 12 and Ballgown 13.

### LncRNA identification

The transcripts overlapping with known mRNAs and transcripts < 200 bp, as well as transcripts with a Coding Potential Calculator (CPC) score 14 < -1 and a Coding-Non-Coding Index (CNCI) score 15 < 0, were removed. Those that were remaining were taken as lncRNAs. The raw and processed data were transmitted to GEO with the GEO accession number GSE132418.

### Differential expression analysis of lncRNAs and mRNAs

The expression values of lncRNAs and mRNAs were calculated as FPKM (fragments per kilobase per million) by StringTie 12. The differentially expressed lncRNAs and mRNAs were selected with fold change ≥2 or ≤0.5 and *P* ≤0.05 using R package-Ballgown 13.

### Quantitative real-time PCR

Quantitative real-time PCR (qRT-PCR) was performed on Applied Biosystems QuantStudio 5 (Thermo Fisher Scientific, USA). Total RNA was extracted from A549, A549/DDP by Trizol (Roche, CA, USA) and reversely transcribed into cDNA by First Strand cDNA Synthesis Kit (Roche, CA, USA). The 2^-ΔΔCT^ methods were employed to analysis the relative expression levels of lncRNAs. The primer sequences were listed in Table 1.

### Functional enrichment analysis of differentially expressed lncRNAs

To clarify the potential functional roles of the dysregulated lncRNAs, the *cis*-target genes of the dysregulated lncRNAs 100 kbp upstream and downstream of the chromosome were picked using a Python script. The neighboring genes were then performed for gene ontology (GO) analysis and Kyoto encyclopedia of genes and genomes (KEGG) enrichment analysis. Significance is expressed as a *P*-value < 0.05.

### Construction of a lncRNA-miRNA-mRNA-related ceRNA regulatory network

First, starBase v3.0 16 and miRcode 17 were employed to analyze the relationships between the dysregulated lncRNAs and miRNAs. starBase v3.0 is a database for studying miRNA-ncRNA, miRNA-mRNA and RNA-RNA interactions from CLIP-seq data 16. miRcode provides miRNA target predictions including lncRNAs, based on the comprehensive GENCODE gene annotation 17. Therefore, we employed miRcode to decipher the interactions between lncRNAs and miRNAs and between miRNAs and mRNAs. Second, miRanda 18, miRcode 17, and TargetScan 19 were employed to decode the relationships between the dysregulated miRNAs and mRNAs. miRanda is a database that can predict miRNA targets and miRNA expression 18. TargetScan is a database that can predict miRNA targets in mammals by matching the conserved 8mer, 7mer, and 6mer sites with the seed region of miRNA 19. Finally, the ceRNA regulatory network was constructed according to the ceRNA regulatory mechanism and the changing trends of lncRNAs, miRNAs and mRNAs, and the network was visualized by Cytoscape 3.6.1.

### TCGA data analysis

The data from the lung adenocarcinoma patients were downloaded from the database of The Cancer Genome Atlas (TCGA, https://cancergenome.nih.gov/). The optimal cut-off point for the expression of all RNAs in the ceRNA regulatory network was divided into high-risk and low-risk groups using the “cutp” function from R package-survMisc. Survival analysis of all RNAs in the ceRNA network was calculated by Kaplan-Meier analysis, which was performed using R package-survival.* P* < 0.05 was considered statistically significant.

### RNA interference experiments

A549/DDP cells were seeded in plates and transfected with 40 nM siRNAs using jetPRIME^®^ (Polyplus-transfection SA, France). SiRNAs were purchased from Shanghai GenePharma Co., Ltd (Shanghai, China). The sense sequence of siTAB2 is 5'-GCUGGGUAUCUCAGUUUAATT-3' and the antisense sequence of siTAB2 is 5'-UUAAACUGAGAUACCCAGCTT-3'. The sense sequence of negative control (siNC) is 5'-UUCUCCGAACGUGUCACGUTT-3'and the antisense sequence of siNC is 5'-ACGUGACACGUUCGGAGAATT-3'. Cells were transfected for at least 24 h before the subsequent experiments.

### Western blot analysis

A549/DDP cells were seeded in 6-well plates at a density of 300,000 cells/well and then transfected with 40 nM siNC or siTAB2 after 24h of cultivation. After 48h of transfection, the cells were lysed using RIPA lysis buffer with protease inhibitor. Total protein (20 μg) was separated on 10% SDS-PAGE, transferred to PVDF membranes (Millipore) and immunoblotted with a rabbit polyclonal antibody against TAB2 (catalog number: 14410-1-AP, Proteintech, USA) or GAPDH (catalog number: 10494-1-AP, Proteintech, USA). Bands were detected by ChemiDoc^TM^ Imaging System (Bio-Rad, USA).

### Growth inhibition assay

Growth inhibition assay was assessed by measuring thiazolyl blue (MTT). A549/DDP cells were seeded in 96-well plates at a density of 4,000 cells/well and then transfected with 40 nM siNC or siTAB2 after 24h of cultivation. Different concentrations of DDP were added to the cells after 24h of transfection, and each group was set in triplicate. After 48h of treatment with DDP, 15μL 5mg/ mL MTT (sigma, USA) was added into the wells and cultured at 37 °C for 4 h. The medium was removed and 100μL DMSO was added into the wells. The plate was shocked and the absorbance was measured at 570 nm using PerkinElmer Multimode Plate Reader EnVision^®^ (PerkinElmer, USA).

## Results

### Differentially expressed lncRNAs in drug-resistant NSCLC cell lines

**Figure 1:**
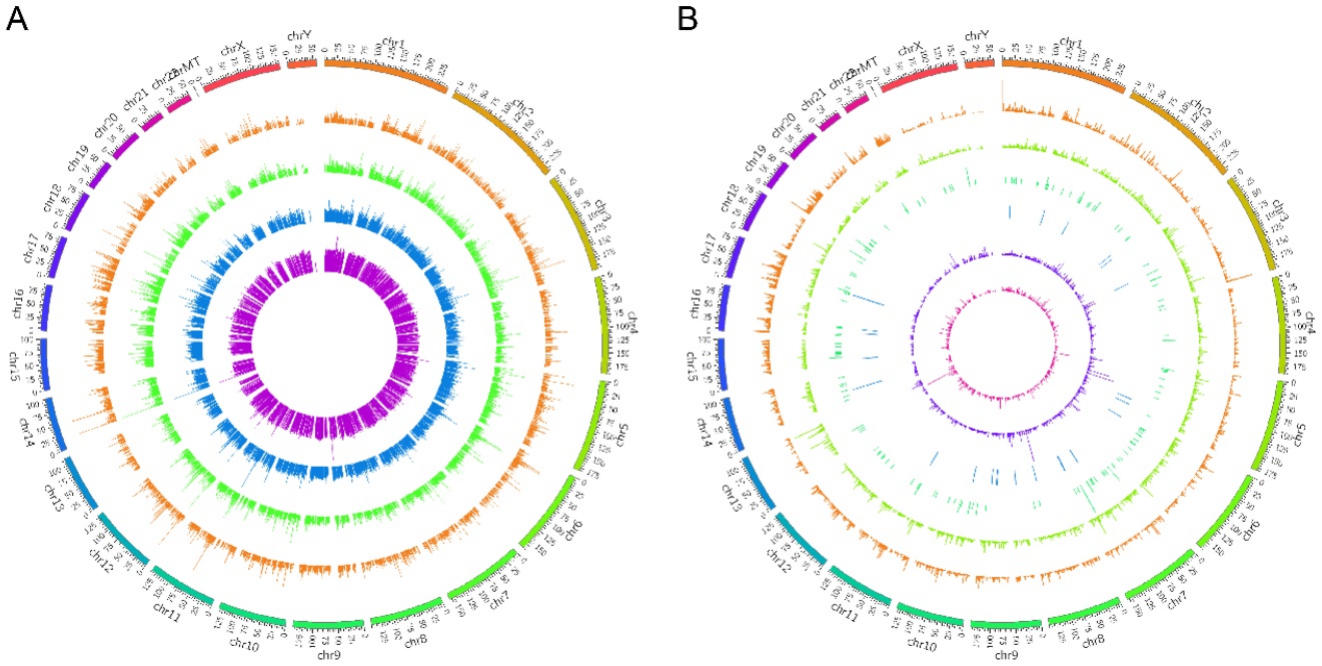
The density distribution of all the lncRNA transcripts on different chromosomes. The global lncRNA abundances on different chromosomes were visualized based on sample expression (**A**) and class code expression (**B**) by mapping all lncRNA transcripts to reference genome.

**Figure 2:**
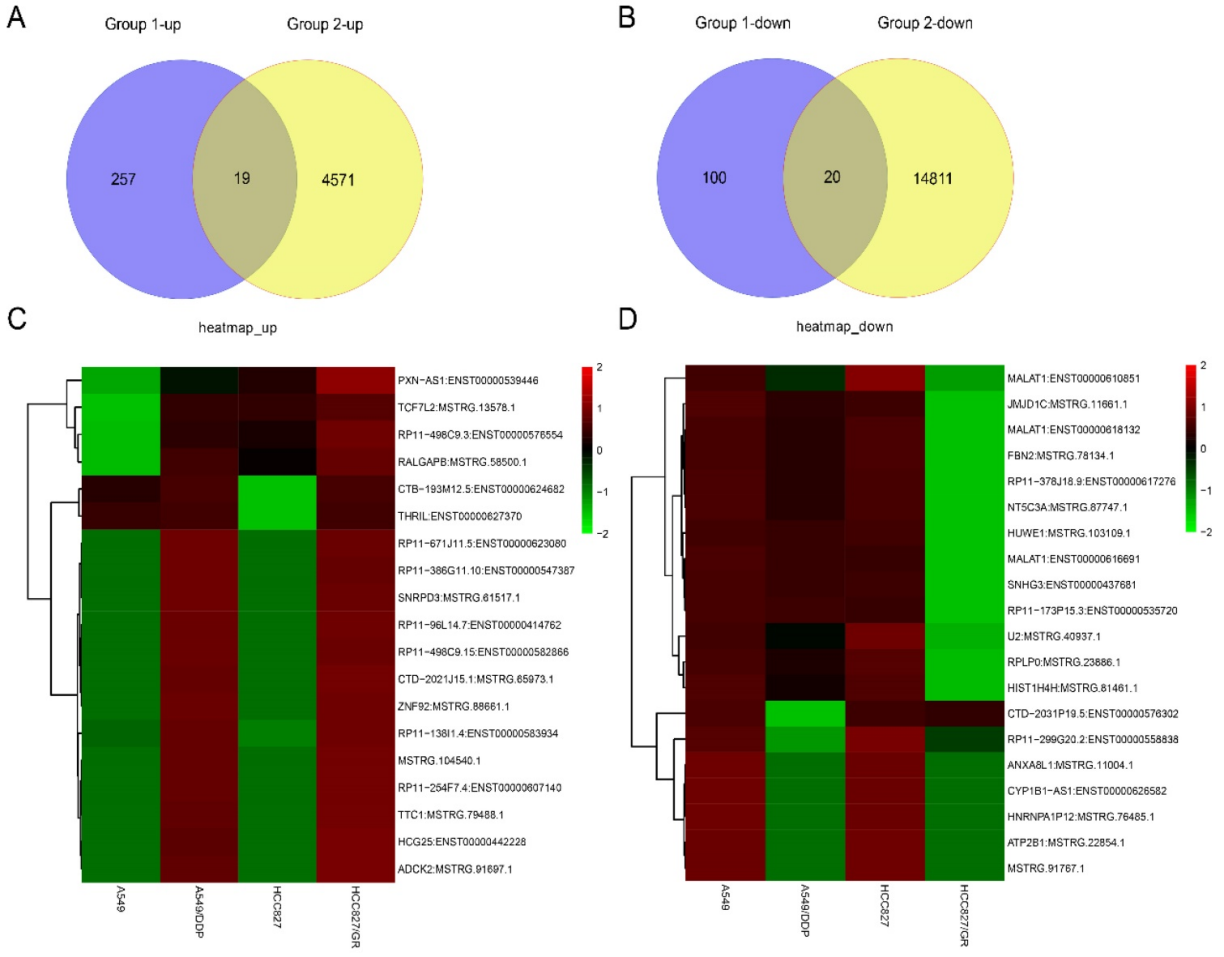
The expression profiles of the same change trends of lncRNAs in drug-resistant NSCLC cell lines. Venn analysis displayed the numbers of the up-regulated lncRNAs (**A**) and the down-regulated lncRNAs (**B**) between group 1(A549/DDP vs A549) and group 2 (HCC827/GR vs HCC827) with the criteria of fold change ≥ 2 or ≤ 0.5 and *P* ≤ 0.05. Heat map showed the expression and hierarchical clustering of the up-regulated lncRNAs (**C**) and the down-regulated lncRNAs (**D**).

RNA sequencing was carried out in the drug-resistant NSCLC cell lines (A549/DDP and HCC827/GR) and their parent cell lines (A549 and HCC827) on an Illumina X10. The global lncRNA abundances on different chromosomes were visualized based on sample expression and class code expression by mapping all lncRNA transcripts to a human reference genome (Fig. 1A and 1B). To identify the dysregulated lncRNAs in those cells, the up- or down-regulated lncRNAs between A549 and A549/DDP cells were taken as one group ,while the up- or down-regulated lncRNAs between HCC827 and HCC827/GR cells were taken as the other group. Then, the same change trends of lncRNAs between these two groups with a fold change ≥ 2.0 or ≤ 0.5 and *P ≤* 0.05 were chosen as lncRNAs candidates. Venn analysis displayed that there were 39 dysregulated lncRNAs, including 19 up-regulated lncRNAs and 20 down-regulated lncRNAs (Fig. 2A and 2B). The heat map analysis shows the expression of the dysregulated lncRNAs visually (Fig. 2C and 2D).

### Validation of lncRNA expression by qRT-PCR

**Figure 3:**
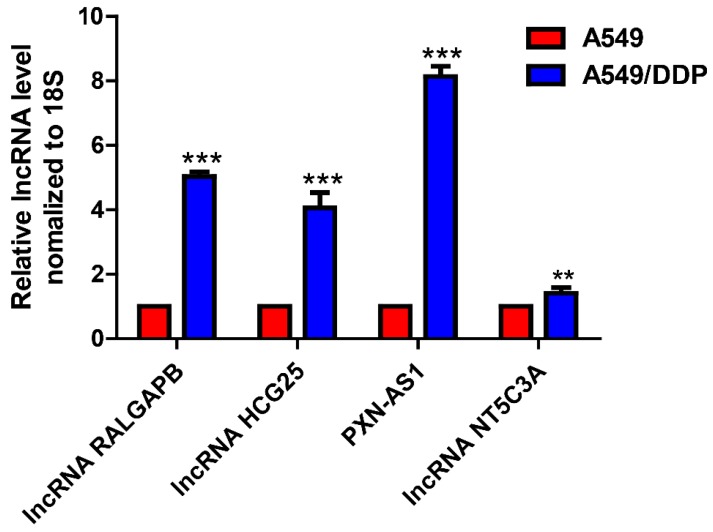
Validation of lncRNA expression in drug-resistant NSCLC cell lines by qRT-PCR.

Four lncRNAs were randomly selected to verify the data of high-throughput RNA sequencing by qRT-PCR. The results showed that the change trends of three of those four lncRNAs were in accordance with the sequence data, suggesting that the sequence data were highly reliable (Fig. 3).

### Functional enrichment analysis of *cis*-target genes of differentially expressed lncRNAs

**Figure 4:**
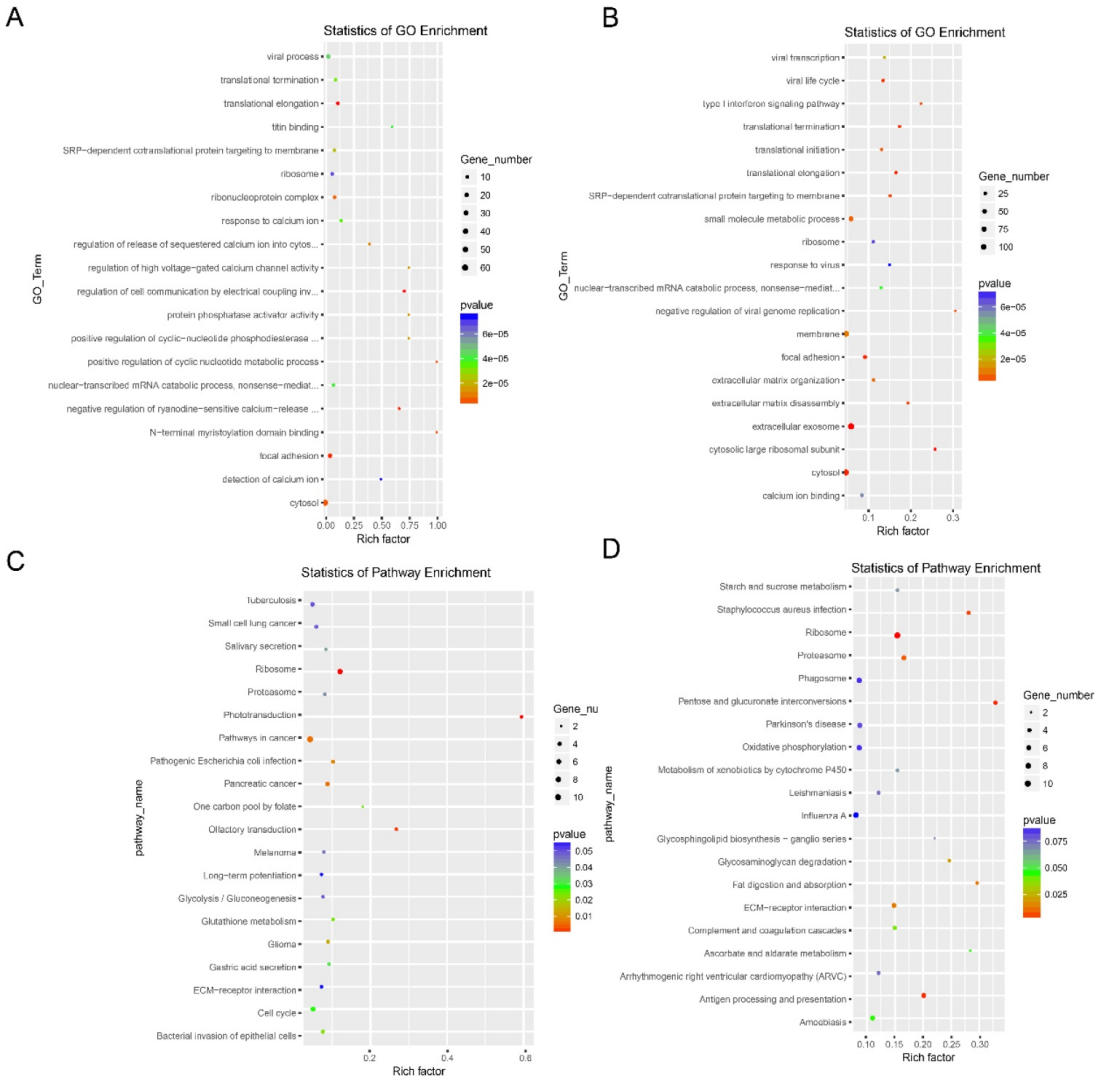
GO enrichment and KEGG pathway analysis of the dysregulated lncRNAs in drug-resistant NSCLC cell lines. GO enrichment analysis of the target genes corresponding to the up-regulated lncRNAs (**A**) and the down-regulated lncRNAs (**B**).KEGG pathway analysis of the target genes corresponding to the up-regulated lncRNAs (**C**) and the down-regulated lncRNAs (**D**).

GO and KEGG pathway analysis were conducted to understand the biological significance and mechanisms of the dysregulated lncRNAs. The *cis*-target genes of the dysregulated lncRNAs upstream and downstream of the chromosome in the range of 100 kbp were selected for GO and KEGG pathway analyses. The main associated GO items of both the up- and down-regulated lncRNAs indicated translational termination, translational elongation, ribosome, and cytosol (Fig. 4A and 4B), suggesting that the dysregulated lncRNAs may be related to the transcriptional regulation of gene expression. All three KEGG pathway analyses of both the up- and down-regulated lncRNAs were ribosome, proteasome, and ECM-receptor interaction (Fig. 4C and 4D).

### Differentially expressed mRNAs in drug-resistant NSCLC cell lines

**Figure 5:**
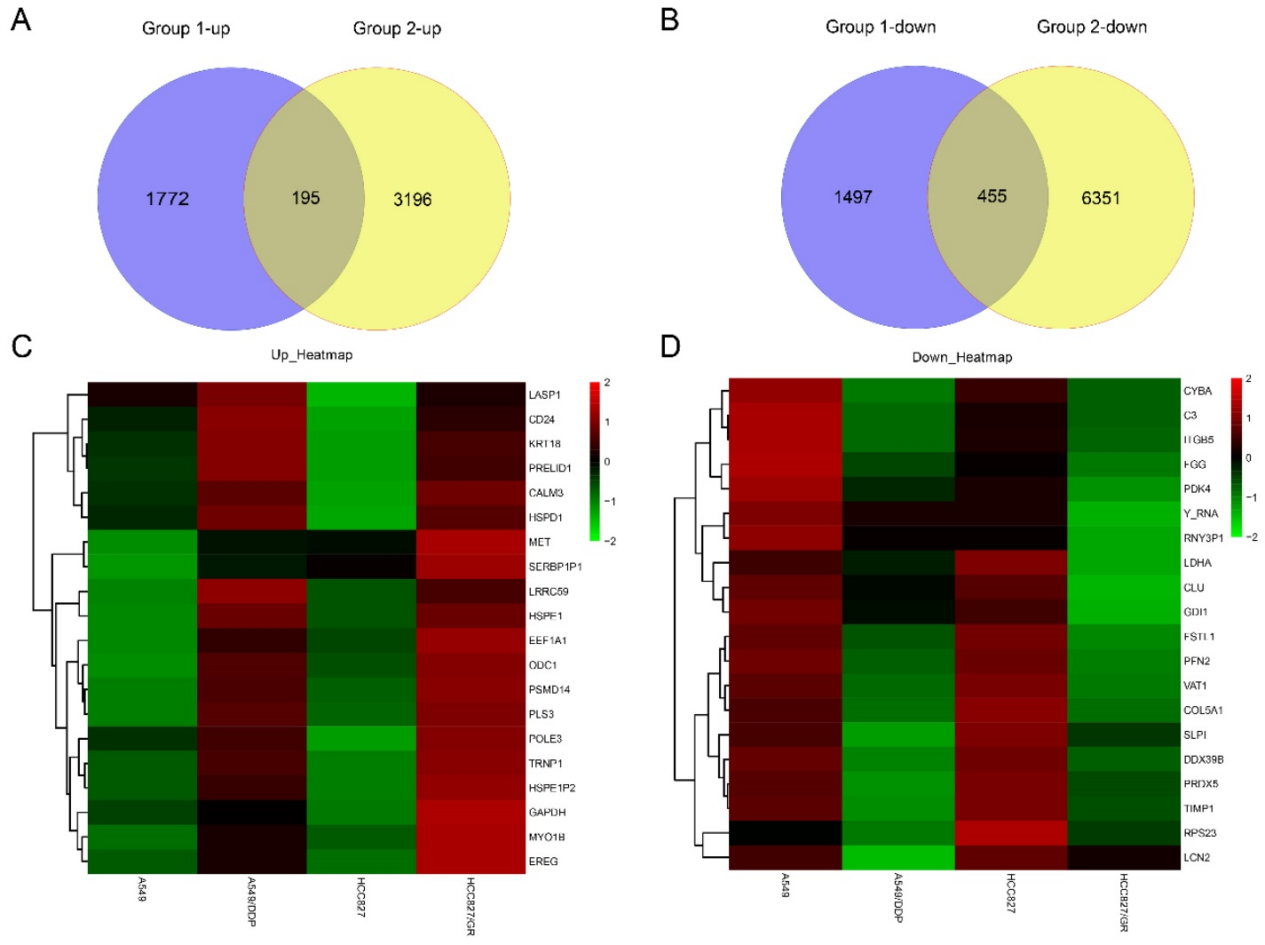
The expression profiles of the same change trends of mRNAs in drug-resistant NSCLC cell lines. Venn analysis displayed the numbers of the up-regulated mRNAs (**A**) and the down-regulated mRNAs (**B**) between group 1(A549/DDP vs A549) and group 2 (HCC827/GR vs HCC827) with the criteria of fold change ≥ 2 or ≤0.5 and P < 0.05. Heat map showed the expression and hierarchical clustering of the first 20 up-regulated mRNAs (**C**) and the first 20 down-regulated mRNAs (**D**).

To investigate the expression levels of mRNAs in drug-resistant NSCLC cell lines, the mRNA expression levels in the drug-resistant NSCLC cell lines (A549/DDP and HCC827/GR) and their parent cell lines (A549 and HCC827) with a fold change ≥ 2.0 or ≤ 0.5 and *P ≤* 0.05 were analyzed. Venn analysis displayed that there were 195 up-regulated mRNAs and 455 down-regulated mRNAs (Fig. 5A and 5B). The heat map analysis shows the expression of the first 20 up-regulated mRNAs and the first 20 down-regulated mRNAs visually (Fig. 5C and 5D).

### Construction of a ceRNA network in drug-resistant NSCLC cell lines based on bioinformatics prediction

To further understand the roles of the dysregulated lncRNAs, miRNAs, and mRNAs in drug-resistant NSCLC cell lines and to clarify the relationships among them, a lncRNA-miRNA-mRNA-related ceRNA regulatory network was done in drug-resistant NSCLC cell lines. The dysregulated miRNA expression levels in the drug-resistant NSCLC cell lines (A549/DDP and HCC827/GR) and their parent cell lines (A549 and HCC827) are described in our previous study 20.

**Figure 6:**
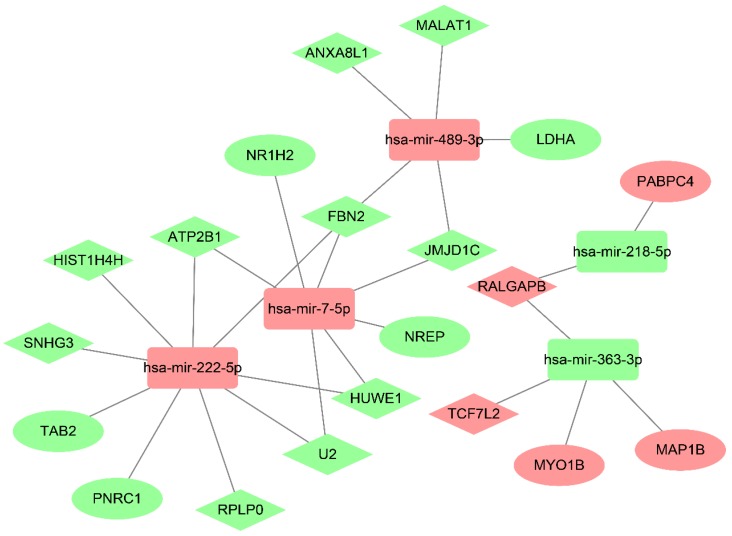
The lncRNA-miRNA‑mRNA ceRNA network analysis in drug-resistant NSCLC cell lines. The lncRNA-miRNA‑mRNA ceRNA network analysis was constructed by Cytoscape. Green and watermelon red represented up-regulated and down-regulated lncRNAs, miRNAs or mRNAs, respectively. Diamond, square and oval represented lncRNAs, miRNAs and mRNAs, respectively.

First, starBase v3.0 and miRcode were employed to analyze the relationships between the dysregulated lncRNAs and miRNAs. Second, miRanda, miRcode, and TargetScan were employed to decode the relationships between the dysregulated miRNAs and mRNAs. And the ceRNA network was constructed in drug-resistant NSCLC cell lines by incorporating 12 lncRNAs (10 up-regulated and two down-regulated), five miRNAs (two up-regulated and three down-regulated), and eight mRNAs (five up-regulated and three down-regulated), according to the ceRNA regulatory mechanism and the change trend of lncRNAs, miRNAs and mRNAs. This was visualized using Cytoscape 3.6.1 (Fig. 6). In the ceRNA network, there were 33 lncRNA-miRNA-mRNA pathways; the result is shown in Table S1.

### Survival analysis of the potential lncRNAs, miRNAs and mRNAs in the ceRNA regulatory network based on TCGA data

**Figure 7:**
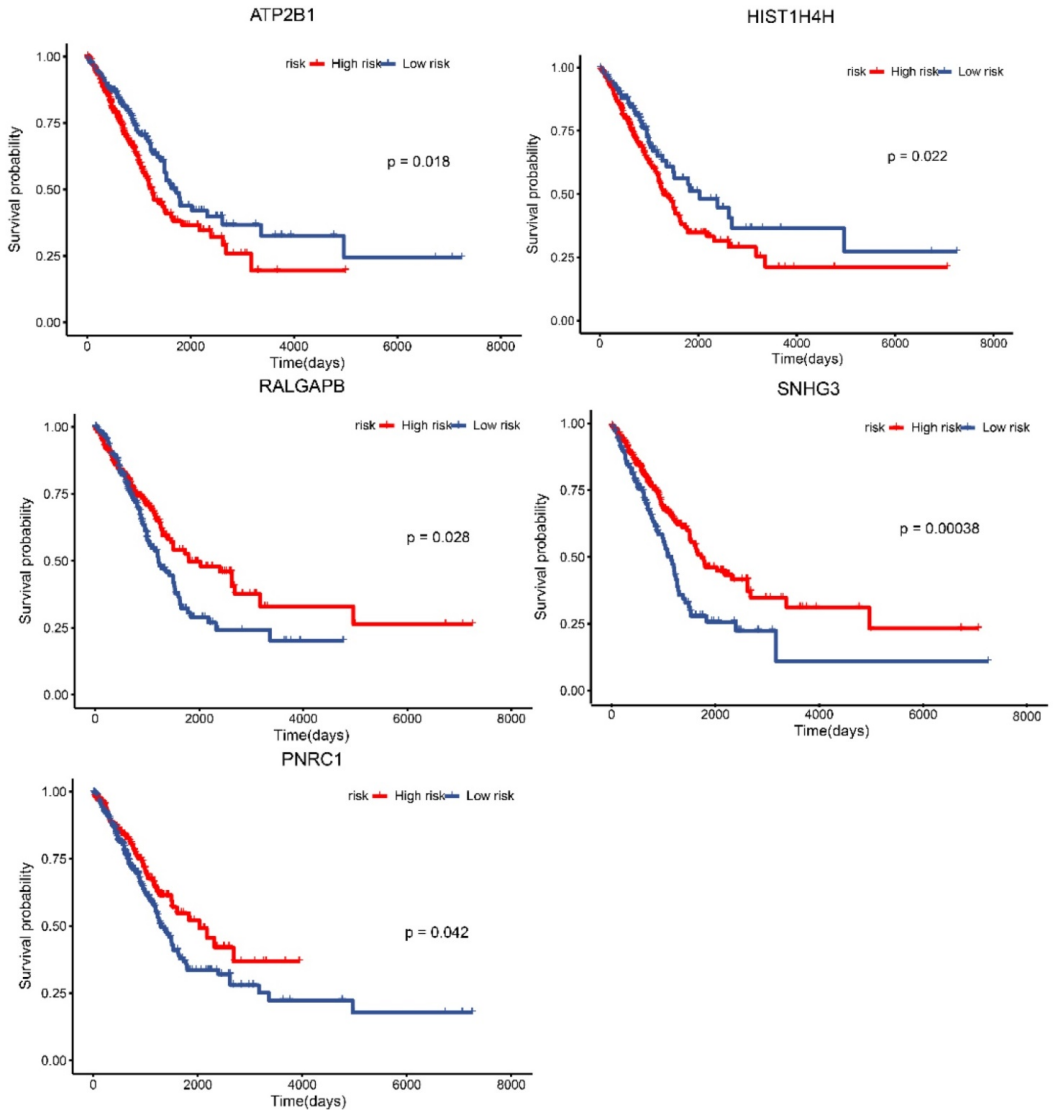
Survival analysis of lncRNAs, miRNAs and mRNAs in the ceRNA network based on TCGA database. Data from TCGA database for lung adenocarcinoma was employed to identify the prognostic characteristics of the promising lncRNAs, miRNAs and mRNAs in the ceRNA network. The results showed the survival analysis of the potential lncRNAs, miRNAs and mRNAs with *P* < 0.05.

TCGA data for lung adenocarcinoma were employed to identify the prognostic characteristics of the promising lncRNAs, miRNAs, and mRNAs in the ceRNA regulatory network. As a result, four lncRNAs and one mRNA were found to be significantly related to overall survival (*P* < 0.05; Fig. 7). Two lncRNAs, lncRNA ATP2B1 and lncRNA HIST1H4H, were negatively correlated with overall survival. Additionally, two lncRNAs, lncRNA RALGAPB and lncRNA SNHG3, and one mRNA, PNRC1, were positively related to overall survival.

### Correlation analysis of the potential lncRNAs, miRNAs, and mRNAs in the ceRNA regulatory network

**Figure 8:**
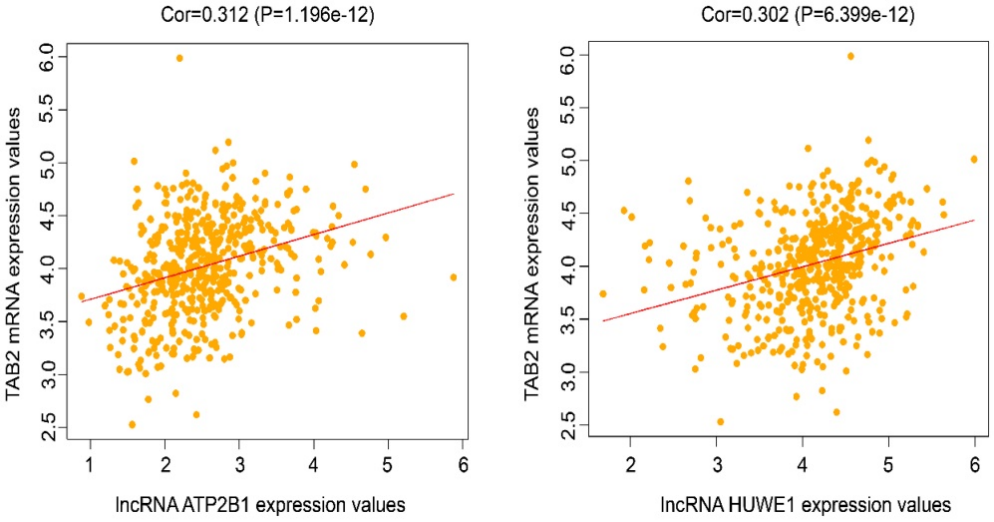
Correlation analysis of the potential lncRNAs and mRNAs in the ceRNA network. Correlation analysis between the expression levels of lncRNAs and their paired mRNAs in lung cancer based on TCGA database was employed. The results showed the correlation analysis of the potential lncRNAs and mRNAs with the correlation coefficient ≥ 0.3 and *P-*value < 0.05.

To further confirm the possible interaction of lncRNAs, miRNAs, and mRNAs in the constructed ceRNA network, correlation analysis based on TCGA data was conducted between the expression levels of 12 lncRNAs and their paired mRNAs in lung cancer. It was shown that lncRNA ATP2B1 and TAB2, and lncRNA HUWE1 and TAB2 had a direct linear correlation with the correlation coefficient ≥ 0.3 and *P-*value < 0.05 (Fig. 8). Combined with the results in Fig. 5, we propose that lncRNA ATP2B1/miR-222-5p/TAB2 and lncRNA HUWE1/miR-222-5p/TAB2 may be potential ceRNA regulatory networks.

### Knocking down TAB2 enhances the sensitivity of drug-resistant NSCLC cell lines

**Figure 9:**
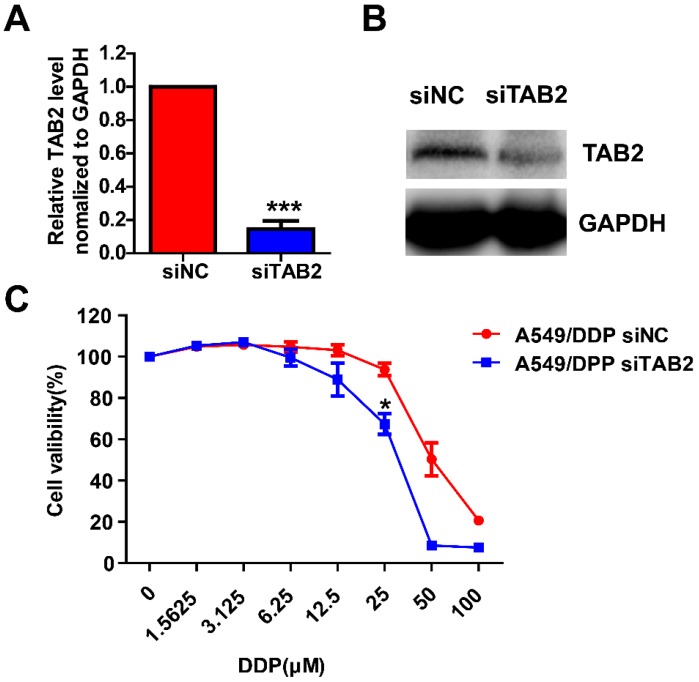
Knocking down TAB2 reduces the sensitivity of drug-resistant NSCLC cell lines. The mRNA and protein levels of TAB2 were detected by qRT-PCR (**A**) and western blot (**B**) after 48h of transfection with siNC or siTAB2 in A549/DDP cells. A549 / DDP cells transfected with siNC or siTAB2 were treated with DDP at different concentrations. The cell growth rate was assessed by MTT, and the OD_570nm_ of A549/DDP cells transfected with siNC but not treated by DDP was taken as 1 (**C**). *P<0.05, ***P<0.001.

To further investigate the roles of TAB2 in the sensitivity of drug-resistant NSCLC cell lines, A549/DDP cells were then transfected with siNC or siTAB2. After 48h, the mRNA and protein levels of TAB2 were reduced as shown in Fig. 9A and 9B. Growth inhibition assay indicated that down-regulation of TAB2 significantly reduced the sensitivity of A549/DDP cells (Fig. 9C).

## Discussion

Drug resistance to chemotherapeutic drugs or targeted medicines is an obstacle encountered in NSCLC treatment 21,22. Besides mRNAs, lncRNAs and miRNAs have been reported to play an important part in the drug resistance of cancers 23,24,25. In fact, some lncRNAs and miRNAs have been demonstrated to take part in drug resistance to chemotherapeutic drugs or targeted medicines of NSCLC, including lncRNA TUG1 related to 5-fluorouracil resistance 26, miR-15b related to sunitinib resistance 27.

Most lncRNAs and miRNAs may function as one member of the ceRNA regulatory networks. In fact, several reports have suggested that the ceRNA networks are related to the occurrence and progression of tumors, such as hepatocellular carcinoma 28, prostate cancer 29 and glioblastoma 30. However, there have been few reports regarding the ceRNA regulatory networks and their involvement in drug resistance except for chemo-esistance to osteosarcoma 23 and cisplatin-resistance to epithelial ovarian cancer 31. In our study, we present a comprehensive perspective on the potential function of lncRNAs and mRNAs. Based on RNA-sequencing and bioinformatics analysis, we constructed a ceRNA network that may be related to drug resistance to chemotherapeutic drugs and targeted medicines in NSCLC. The selected lncRNAs, miRNAs, and mRNAs in the networks corresponded with the ceRNA rule. There were 33 lncRNA-miRNA-mRNA pathways in the ceRNA regulatory network, includng 12 lncRNAs, five miRNAs and eight mRNAs. In addition, we conducted survival analysis and correlation analysis of the potential lncRNAs, miRNAs and mRNAs in the ceRNA regulatory network using TCGA data and found that four lncRNAs (lncRNA ATP2B1, lncRNA HIST1H4H, lncRNA RALGAPB, and lncRNA SNHG3) and one mRNA (PNRC1) were significantly related with overall survival. In addition, lncRNA ATP2B1/miR-222-5p/TAB2 and lncRNA HUWE1/miR-222-5p/TAB2 were revealed as potential ceRNA regulatory networks.

There have been a few reports suggesting that miR-222-5p and TAB2 are related to the occurrence and progression of tumors or drug resistance. MiR-222-5p has been reported to be involved in triple-negative breast cancer 32, central lymph node metastases of papillary thyroid cancer 33, ovarian cancer 34, and resistance tamoxifen treatment in breast cancer 35. TAB2 is an activator of MAP3K7/TAK1 and is required for TLR-mediated or IL-1-induced NF-kB activation 36,37. The NF-kB signaling pathway is a key regulator of tumor occurrence and development and can also take part in drug resistance to chemotherapy and targeted therapy 38,39. Additionally, TAB2 has been reported to serve as a new target for tamoxifen resistance in breast cancer 40, and down-regulation of TAB2 may sensitize NSCLC cells to BMS-690514, a new pan-HER/VEGFR inhibitor 41. In our study, we found that down-regulation of TAB2 enhanced the sensitivity of A549/DDP cells and we proposed that TAB2 might play a vital role in drug resistance to chemotherapy and targeted therapy in NSCLC. Until now, there have been no reports regarding TAB2 in NSCLC drug resistance, and further experiments are necessary to evaluate the role and mechanism of TAB2 in NSCLC drug resistance.

The mechanisms behind resistance to chemotherapeutic drugs or targeted medicines may differ greatly, but it is anticipated that some overlapping mechanisms between these two distinct types of drug resistance may exist. For example, ECM, which plays a vital role in biological processes such as cell proliferation, differentiation, and adhesion 42, can also be associated with chemotherapy resistance 43, 44 and may be related to gefitinib resistance via the ECM receptor 45. Interestingly, we found that ECM-receptor interaction was one KEGG pathway in both the up- and down-regulated lncRNAs. This discovery further confirmed our speculation and may deepen our understanding of drug resistance.

To our knowledge, this is the first report to construct and analyse the ceRNA network of lncRNAs, miRNAs and mRNAs in NSCLC drug resistance to chemotherapeutic drugs or targeted medicines. However, there are some limitations to our study. For example, the validation of potential lncRNAs, miRNAs, and mRNAs in drug resistance of NSCLC were not performed, and the mechanism of the ceRNA network was not confirmed by luciferase reporter assay or RNA immunoprecipitation assays.

## Conclusion

In conclusion, we provide here a comprehensive expression profile of lncRNAs, miRNAs, and mRNAs. We also constructed a lncRNA-miRNA-mRNA ceRNA regulatory network for drug resistance to chemotherapeutic drugs or targeted medicines in NSCLC. In addition, we found that lncRNA ATP2B1/miR-222-5p/TAB2 and lncRNA HUWE1/miR-222-5p/TAB2 are potential ceRNA regulatory networks in NSCLC drug resistance according to TCGA data and bioinformatics analysis. These results revealed that the lncRNA-miRNA-mRNA ceRNA regulatory network might play a vital role in drug resistance to NSCLC and might be a promising therapeutic strategy for further study. These results will deepen our comprehension of the ceRNA mechanisms involved in drug resistance to NSCLC.

## Supplementary Material

Supplementary table.

## Figures and Tables

**Table 1:** The primers for qRT-PCR

Name	Sequence (5'-3')
lncRNA RALGAPB (F)	TGAAGCCATTGTTGGTTGGC
lncRNA RALGAPB (R)	AGGGTCTTAAGGGTCTTTACCA
lncRNA HCG25 (F)	CAGGAAAGGAGGGTGACAGAC
lncRNA HCG25 (R)	GCTTTGGTAGTTCCTGCCTTCA
PXN-AS1 (F)	TGGCGAGCTCAGCAAACTAA
PXN-AS1 (R)	TTTGCGTGCTTCCTCTTTGC
lncRNA NT5C3A (F)	AAGGAGGCTTCACTGGGACT
lncRNA NT5C3A (R)	GGTCAACGTAGGCACCTCTAA
TAB2 (F)	CTCCTGGTGGTACAACTCGAC
TAB2 (R)	TGATTTGGCTGTTGAGATGAGG
18S (F)	GTAACCCGTTGAACCCCATT
18S (R)	CCATCCAATCGGTAGTAGCG
GAPDH (F)	TGTTGCCATCAATGACCCCTT
GAPDH (R)	CTCCACGACGTACTCAGCG

## Abbreviations

ceRNA: competing endogenous RNA
GEO: gene expression omnibus
GO: gene ontology
KEGG: kyoto encyclopedia of genes and genomics
lncRNA: long non-coding RNAs
miRNA: microRNA
MRE: miRNA-response element
NCBI: national center for biotechnology information
NSCLC: non-small cell lung cancer
qRT-PCR: quantitative real-time PCR
TCGA: the cancer genome atlas
